# Assessing site signal preservation in reference chronologies for dendro-provenancing

**DOI:** 10.1371/journal.pone.0239425

**Published:** 2020-09-24

**Authors:** Urs Gut

**Affiliations:** 1 Fachbereich Prähistorische Archäologie, Institut für Archäologie, University of Zurich, Zurich, Switzerland; 2 Forest Ecology, Institute of Terrestrial Ecosystems, Department of Environmental System Science, ETH Zurich, Zurich, Switzerland; University of Nevada, Reno, UNITED STATES

## Abstract

Regional differences in tree growth can be used to approximate the geographical provenance of ring-width series (‘dendro-provenancing’). This method relies on cross-dated ring-width series (reference chronologies) that are thought to represent the radial growth signal of trees in a given region. Reference chronologies are often established from ring-width series of living tree populations. Frequently, they are too short to allow for investigating the provenance of historical wood. Thus, references are extended by ring-width series from buildings and art-historical objects that exhibit best matching growth patterns with the living tree references. Yet, series from other provenances may erroneously be included. Thereby the local or regional growth signal of the references is progressively contaminated, but this has received little attention to date. I investigate this contamination risk using a simulation approach that allows for generating pseudo site chronologies that preserve the relevant statistical properties of the real site chronologies. While the exact provenance of historical wood is unknown, for simulated ring-width series the provenance is unambiguous. Hence, pseudo reference chronologies may be established while monitoring the signal mixture. Specifically, 15 site chronologies of Norway spruce (*Picea abies* (L.) H. Karst.) from northeastern Switzerland were used to generate 15 pseudo site growth signals that span 1000 years. The simulation demonstrates that quasi uncontaminated references can be established in ideal circumstances for the study area. However, the thresholds for the similarity in between-series correlation must be very high. Even then, contaminated pseudo references occurred in rare cases during the simulation. Yet, elevation-specific pseudo references were established with lower thresholds. Simulation currently offers the only approach for assessing the contamination risk of reference chronologies, and it allows for elucidating the conditions under which acceptable levels of contamination can be guaranteed. Therefore, the present approach paves the way towards a practical simulation tool for dendro-provenancing.

## 1 Introduction

Dendrochronology (most commonly, the study of tree-ring width) is used in many scientific disciplines, e.g. in Forestry, Climatology and Ecology [1]. In Archaeology and History, it is among the most important methods for establishing absolute chronological frameworks and providing environmental information [2–4].

Dendro-provenancing goes beyond the dating of wooden objects and capitalizes on regional differences in tree growth that originate from regional differences in climatic and site conditions. If such differences are pronounced, dendro-provenancing can be used to approximate the site of growth of trees that were used to produce wooden objects and artifacts [5–8]. Thus, for example, timber trade routes and areas of timber supply of cities can be reconstructed [9, 10], or the origin of wooden resources used e.g. for panel painting and shipbuilding can be determined [11–13].

The potentials and limitations of dendro-provenancing have been reviewed [14, 15], and the key assumptions underlying dendro-provenancing have been formulated and evaluated as well [15]. In short, dendro-provenancing is based on the comparison of candidate ring-width series from historical objects of unknown provenance to a framework of local reference chronologies. The reference chronologies are established from cross-dated samples of ring-width measurements that are thought to represent local tree-growth. The location of the provenance is then approximated by the geographical area in which best matches between the ring-width patterns of a candidate series and the reference chronologies are found [16]. Generally, best matches are determined statistically using the same methods as for the (cross-)dating of ring-width series [17, 18].

In a previous study [15], ring-width chronologies were established from living spruce (*Picea abies* (L.) H. Karst.) at 15 sites in northeastern Switzerland. The respective dataset provides the basis for reference chronologies for dendro-provenancing. Specifically, the living tree chronologies can be extended by best matching historical ring-width series. Indeed, the dataset showed sufficient between-site year-to-year (high-frequency) ring-width variability to potentially allow for dendro-provenancing in the study area [15]. However, the extent to which these differences are preserved when living tree chronologies are extended by successively adding best matching historical ring-width series has received little attention to date [18–21]. For dating, the merging of site growth signals is relatively inconsequential, because the supra-regional climatically determined growth signal is preserved [22]. However, for dendro-provenancing to work, reference chronologies must preserve local to regional growth signals.

In most studies, historical reference chronologies do not incorporate living tree chronologies [5–14, 18–21]. Thus, investigators need to assume that their reference chronologies represent a–however loosely defined–regional growth signal, which is uncontaminated. Obviously, without references that represent a geographically characteristic signal, the results of dendro-provenancing are spurious [15]. Unfortunately, the preservation of local and regional growth signals cannot be studied based on historical ring-width series because their geographical provenance often is unknown. At best, the site of growth may be approximated by watersheds [9]. This is applicable to the pre-industrial epoch only, i.e. before the onset of timber trade via railroads. Even earlier, rafting of timber sometimes covered long distances [10, 23]. Thus, only in areas where past timber trade was spatially limited there is a chance for reference chronologies to capture local growth signals [24]. However, the establishment of such chronologies is extremely time- and labor-intensive. In addition, if the between-site signal differences are unpronounced, the different local references probably merge into a single supra-regional chronology. In studies that have a large geographical scale, such chronologies can sometimes still be useful to roughly estimate the provenance [11, 12, 14]. However, if the geographical resolution and the reliability of dendro-provenancing are to be improved, the potential for establishing uncontaminated reference chronologies needs to be assessed prior to field work (i.e. prior to the establishment of historical references) and prior to the determination of provenance.

Aside from living tree chronologies, only for simulated series the site origin is retraceable. Moreover, the length of living tree chronologies is biologically limited. In simulation, however, pseudo historical series of arbitrary length can be generated whose site provenances are known. Consequently, the contamination risk can be monitored at a greater temporal depth when pseudo reference chronologies are established. Thus, an evaluation of signal preservation in reference chronologies is best approached via simulation.

The basic concept underlying this paper can be summarized as follows: Firstly, the most relevant statistical properties of recently (i.e., in 2015 and 2016) collected site chronologies are characterized and an adequate simulation model is developed. Secondly, this model is used to generate a set of simulated historic pseudo ring-width series. The latter are samples from an underlying set of pseudo site chronology signals that preserve the covariance of the original site chronologies. Thus, the (dis-)similarities of growth that exists between the original chronologies is reflected in the simulation. Thirdly, once the pseudo historical tree-ring data have been generated, an algorithm is used that mimics the extension of site chronologies by adding best matching pseudo historical series. Finally, the composition of the generated pseudo reference chronologies is evaluated. The critical point is that the site provenance is known for all pseudo historical series, as they were sampled from the respective underlying pseudo site signals. Hence, it is possible to examine the composition of the pseudo references and determine the degree of signal mixture. The current case study is limited to the material gathered earlier [15], but the approach is transferable to other dendro-provenancing studies.

The objectives of this study relate to the simulation model, the classification algorithm and the evaluation of the algorithmically generated pseudo reference chronologies, and are as follows:

1. To introduce and evaluate a simulation model for generating pseudo historical ring-width chronologies.
2. To develop an algorithm that mimics the process of establishing reference chronologies for dendro-provenancing.
3. To examine the signal composition of the pseudo reference chronologies and assess the potential for establishing real reference chronologies in the study area.

## 2 Materials and methods

### 2.1 Real ring-width dataset

Real ring-width datasets are needed to initialize the simulation. The dataset used for this purpose comprises 401 ring-width series that represent a set of 15 site chronologies of Norway spruce (*Picea abies* (L.) H. Karst.) with a replication of 15 to 32 ring-width series per site [15].

Further details on the sampling design, measurement, cross-dating and meta data are given in [15]. The ring-width dataset is provided in S1 File (R package data *chrono*.*rwl* in S1 File).

For the simulation, the actual site name is unimportant. Hence, only abbreviations are used to label the pseudo site signals. However, the letters preceding the dot in the abbreviations (e.g. as used in subsection ‘Pref composition’) are identical to the real site abbreviations introduced in [15]. Thus, the pseudo sites signals can be traced back to their underlying real site signals for more detailed scrutiny.

### 2.2 Simulation

All calculations and statistical analyses were done in R (3.6.0) [25]. Specifically, an *ad hoc* R package was written to perform the simulation. The complete code can be found in S1 File.

**Fig 1:**
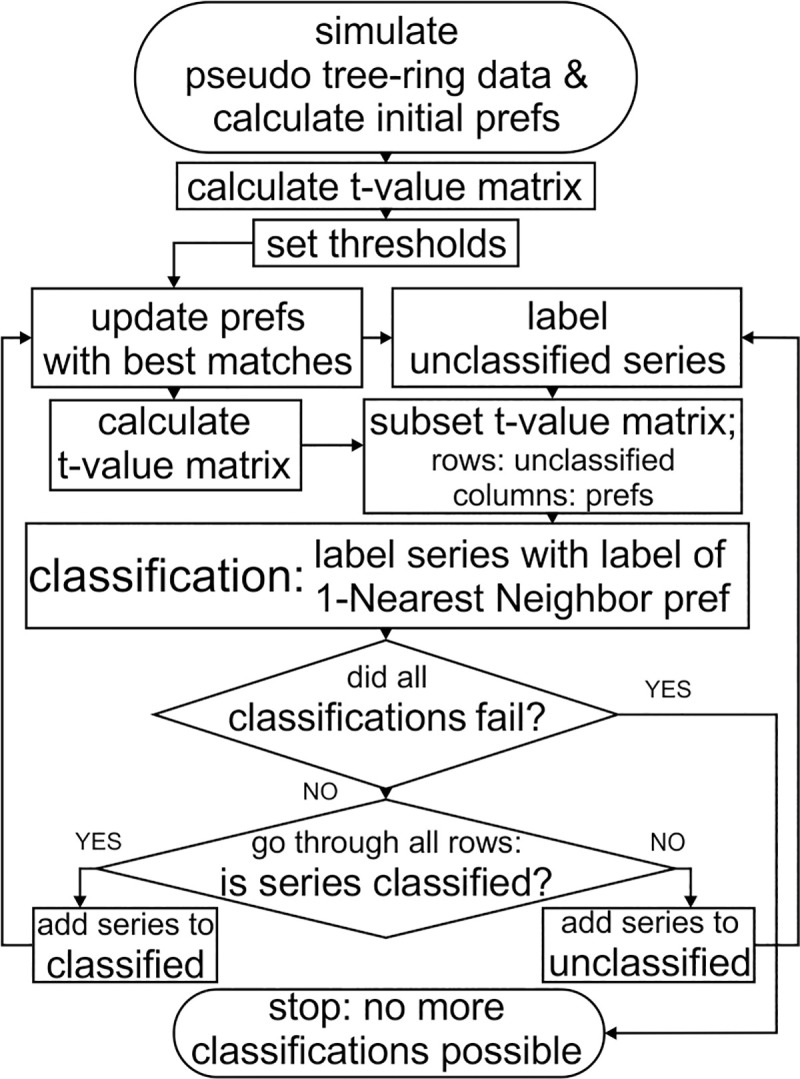
Flowchart of the PREF-Constructor algorithm.

Auto-regressive (henceforth: ar) residual series effectively preserve the signal that is relevant for dendro-provenancing [15]. Thus, ar residual chronologies were used for the simulation. Unlike in raw ring-width series, in ar residuals the medium- (5–15 years) and low-frequency (>15 years) growth variability is quasi absent [26]. The individual ar residual series hence represent the year-to-year (high-frequency) growth variability of each individual tree ring-width series. Thus, for each year of an ar chronology, there is a sample equal to the number of trees that were sampled and that featured a ring in the respective year (Fig 1A in S2 File). These yearly distributions of ar residuals may be approximated by normal distributions, as, theoretically, ar residuals are series of normally distributed random shocks [27, 28]. Hence, as for any other normally distributed variable, the yearly means and standard deviations of the theoretical population distributions of the ar residuals can be estimated by calculating the sample means and standard deviations of the empirical distributions of the ar residuals [28].

Thus, as a first step towards a simulation model of the real tree-ring data, for each of the 15 raw ring-width chronologies an auto-regressive residual chronology was calculated using the default settings of the detrender-function in the R package *dplR*, i.e. by fitting an auto-regressive model to the raw ring-widths and choosing the model that minimized Akaike’s Information Criterion [29, 30]. This choice of preprocessing was supported by the high classification performance of this method in the study of [15]. When requiring a minimal replication of 15 data points per year of the chronology, the 15 resulting ar chronologies covered a common period of 63 years (i.e., from 1951 to 2014). Subsequently, series of empirical mean values and standard deviations were calculated for these 63 years. This resulted in a 63 × 30 matrix containing 15 column vectors of yearly mean values (rmv) and 15 column vectors of yearly standard deviations (rsd).

To sidestep the limited length of the real dataset, a 1000 × 30 matrix was generated whose columns had zero covariance and followed a standard normal distribution (Fig 1B in S2 File). This matrix was transformed to a matrix with the same scale and covariance as the real data matrix. The transformation matrix was generated by decomposing the covariance matrix of the real rmv-rsd matrix via its eigenvalues [31, 32].

The R implementation of the function *rmvnorm* (MASS package) assumes a multivariate normal distribution of the underlying variables [32]. Thus, the transformation was valid for covariance matrices calculated from such variables only. For the rmv vectors, a multivariate normal distribution was reasonable to assume because the indices of the ar residuals were themselves normally distributed [27, 28]. For the rsd vectors, however, a multivariate log-normal distribution seemed more appropriate (Fig 1 in S2 File). Hence, the rsd were log-transformed (natural logarithm) prior to the calculation of the covariance matrix, whose decomposition was used to linearly transform the uncorrelated random data matrix (Fig 1B in S2 File).

After the linear transformation, the generated matrix contained 30 column vectors of length 1000, with 15 columns for the yearly pseudo mean values (pmv) and 15 for the logarithms of the yearly pseudo standard deviations (ln(psd)) of each pseudo chronology. Next, the ln(psd) were raised to their power to provide the yearly standard deviations of the pseudo chronologies (psd). Finally, the resulting matrix contained all measures of central tendency and dispersion necessary to parameterize the respective sequences of yearly normal distributions for each pseudo site chronology (cf. matrix *P*, Fig 1B in S2 File).

#### 2.2.1 Pseudo historical series

To generate individual pseudo series (ps), random samples were sequentially drawn from the sequences of normal distributions defined by the parameters in the pseudo signal matrix. Thus, ps with a length of 1000 years were generated. The ps were cut into partitions (pseudo historical series, phs) that were of similar lengths as the series found in a typical dataset of historical ring-width series. Here, the dataset of the Dendrochronological Laboratory of the City of Zurich was used to determine the partitioning (R package data *distr*.*sl* in S1 File).

To partition a 1000 years ps, series lengths were drawn randomly from the pool of series lengths of *Picea abies* (length ≥ 50 years) until the sum of the drawn series lengths was ≥ 1000 years. Then the 1000 years ps was cut into subseries. Often, this resulted in the last partition being shorter than 50 years, which were discarded during later analyses (cf. section PREF-Constructor Algorithm, below). The sampling of ps and their subsequent partitioning into phs was repeated until a replication of 30 phs per year and pseudo chronology was achieved.

#### 2.2.2 Pseudo object chronologies

Generally, object-based chronologies are the core components of reference chronologies for dendro-provenancing rather than single tree-ring series. Such object-based chronologies already represent an aggregate (i.e. the mean) of several ring-width series, which were sampled in historical objects (e.g. the roof beams of a building). If timber sources for the construction of respective objects were spatially limited, object-based chronologies represent mean local tree growth [10]. To allow for a comparison of pseudo reference chronologies established from phs and pseudo reference chronologies generated from pseudo object chronologies (poc/pocs), the above procedure of generating phs was slightly modified, as described below.

Instead of drawing a single ps, 6 ps were sampled from the sequence of yearly normal distributions of a respective pseudo chronology signal. This replication was chosen arbitrarily but seems to be a realistic assumption for the average number of samples taken per historical object in a real setting [33]. Subsequently, the 6 ps were averaged prior to the partitioning (as described in the previous section). Thus, the resulting pocs represent an object chronology with a replication of 6 individual ps per year each. This reflects an ideal scenario, in which the object chronologies are mean value chronologies calculated from phs that originated from the same pseudo site signal. Again, as with the phs approach the sampling and partitioning was repeated until a replication of 30 pocs per year and pseudo chronology was achieved.

In a real setting, there is a chance for historical building chronologies to contain a mixture of different site signals [10]. Thus, some simulations were done with pocs containing a mixture of ps originating from different pseudo signals. Specifically, the ratio of ps originating from the correct, ‘local’ pseudo signal was lowered. Thus, this so-called on-site ratio (osr) was lowered from 1 (all 6 ps on-site) to 0.83, i.e. 1 of the 6 ps originates from an off-site pseudo signal, to 0.67, i.e. 2 of the 6 ps are off-site ps.

### 2.3 PREF-Constructor algorithm

To mimic the establishment of reference chronologies by adding historical ring-width series to initial living tree chronologies and automate this process, a Pseudo Reference Constructor Algorithm (PREF-Constructor) was developed and implemented in the *ad hoc* R package mentioned above (S1 File). The algorithm generates pseudo reference chronologies (pref/prefs) by adding phs/pocs to the best fitting initial pref, i.e. to the One-Nearest-Neighbor chronology [34–36]. Thus, prefs are established that cover 1000 years in ideal circumstances (Fig 1).

The 15 initial prefs of this study represent the 15 mean values calculated from 30 ps of length 150 years per pseudo site chronology. These 30 ps per initial pref were sampled in addition to the already simulated pseudo dataset. This mimics a scenario in which a dendrochronologist starts out with 15 unmixed mean site chronologies (the initial prefs) and extends these by adding the best matching series from the hypothetical laboratories’ historical dataset (here, the simulated phs/poc dataset).

Best matches were determined statistically, i.e. by calculating a t-value for each Pearson’s correlation coefficient that resulted from pairwise comparisons between a candidate series (phs/poc) and the 15 initial prefs [37, 38]. This method in combination with ar prewhitening had a high performance in a previous study [15]. Because here the ps resemble ar residual series, a comparable performance was expected.

Potential best matches were required to fulfill threshold criteria. Specifically, the thresholds for t-values that were tested were t ≥ 5 (t5), t ≥ 10 (t10), t ≥ 15 (t15) and t ≥ 20 (t20) at a minimum overlap of 50 years. If several matches fulfilled the threshold criteria, the respective candidate series was classified to the best matching initial pref, i.e. the chronology that yielded the highest t-value for the pairwise comparison.

In a real dataset, potential cross-dating errors complicate the classification. The PREF-Constructor assumes that the cross-dating of each candidate phs/poc is correct. Thus, best matches need to be found for one correlation position only.

Once the best matching initial pref had been determined for all phs/pocs, the series were labelled (i.e. classified) according to the label of the best matching initial pref. After this initial run, the first prefs were established from the classified phs/pocs. Subsequently, the phs/pocs that were yet unclassified were compared to the prefs just established. Again, all series that fulfilled the threshold criteria were classified and added to the respective pref. Series that remained unclassified were checked again during later runs, after the establishment of the prefs had advanced. Thus, the algorithm proceeded until all series were classified, or until no unclassified series fulfilled the threshold criteria anymore, i.e. no classifications were possible any more (Fig 1).

### 2.4 Evaluation

One thousand pseudo datasets comprising 15 pseudo chronologies each were generated to evaluate 1) the simulation model and 2) the PREF-Constructor results. This 1000-fold repetition of the simulation was done for the phs approach as well as for the poc approach and with different settings for the thresholds. Thus, the variability in pref composition that existed between the different repetitions due to random sampling was quantified to avoid unfounded interpretations based on the results of a single simulation repetition.

In the following two subsections, performance indicators are introduced that provide a basis for quantifying the stability of the pseudo signals across all repetitions. Moreover, methods are presented that allow for the comparison of PREF-Constructor classifications between different simulation settings.

#### 2.4.1 Stability of the pseudo signals

The simulated pseudo signals must remain stable across different simulation repetitions. Instability would indicate that the individual pseudo series (ps) were sampled from a different set of pseudo site signals in each repetition of the simulation. This would render the 1000 pseudo datasets incomparable. Thus, the stability of the intercorrelation between the pseudo mean values (pmv) or the sample pseudo mean value chronologies (spmv), respectively, was investigated.

*Δ{pmv-rmv}*. To quantify the stability of the linear transformation that yielded the pseudo mean values (pmv/pmvs) for the simulation, the pairwise correlations (i.e., the correlation matrix) between the pmv vectors were calculated for each of the 1000 simulation repetitions. Subsequently, the correlation matrix for the mean values of the real site chronologies (rmv/rmvs) was calculated, and the range of differences between the entries of the correlation matrices calculated for the pmvs and for the rmvs (Δ{pmv-rmv}) was determined.

*Δ{spmv-rmv}*. The linear transformation that yielded the pmvs was expected to be stable, except for rounding errors [31]. However, more instability was anticipated for the mean values calculated from phs chronologies. Even if the PREF-Constructor algorithm were to classify each phs correctly, the pseudo reference chronologies (pref) calculated from these phs would not perfectly reproduce the intercorrelation of the pmv. This was to be expected because the phs are random realizations of the respective pseudo signals (section Simulation). Thus, the variability introduced due to random sampling needed to be quantified. Similar to Δ{pmv-rmv}, the Δ{spmv-rmv} indicates the range of differences between the correlation matrix entries of the rmv and the sample pseudo mean value chronologies (spmv) of each of the 1000 simulation repetitions. Thus, the spmv are the hypothetical mean value chronologies that result from averaging the phs according to a perfect classification. No further investigations were done on the stability of the poc chronology signals because no further instability results from averaging phs into pocs (for an on-site ratio of 1).

#### 2.4.2 Pref composition

Pref composition was investigated by calculating the percentage of generated series that were classified during each simulation repetition. Of these classified series, the percentage of correctly classified series was determined. To investigate the dynamic establishment of the prefs, the off-site poc/phs contamination was calculated for each run of classifications executed by the PREF-Constructor (section PREF-Constructor Algorithm). That is, the percentage of phs/pocs originating from a pseudo site signal other than the pseudo signal that had been used to initialize the respective pref was calculated in every run.

Hypothetically, while the generated prefs may represent mixtures of pseudo site signals they may still carry watershed and/or elevation-specific pseudo signals. Therefore, the phs/pocs were grouped according to contrast groups other than pseudo site provenance. Subsequently, the off-contrast contamination was calculated, i.e. the percentage of phs/pocs originating from a contrast group other than the group that the initial pseudo signal of the respective pref was attributed to. To define these contrast groups, the elevation bands and watersheds/regions of the real site chronologies [15] were used to group the different pseudo site signals:

1. Elevation contrasts (site abbreviations refer to names in [15]; cf. section Real ring-width dataset):
  - Low: real site located < 1000m a.s.l. (sw.pseudo, hw.pseudo, bw.pseudo, ew.pseudo, fri.pseudo, nb.pseudo, sb.pseudo)
  - Medium: real site located 1000-1500m a.s.l. (chw.pseudo, kar.pseudo, how.pseudo, gw.pseudo, gand.pseudo)
  - High: real site located > 1500m a.s.l. (furg.pseudo, rw.pseudo, ww.pseudo)
2. Watershed contrasts:
  - Sihl (pseudo sites: fri.pseudo, furg.pseudo, gw.pseudo, kar.pseudo, sw.pseudo).
  - Linth (gand.pseudo, how.pseudo, hw.pseudo, nb.pseudo, rw.pseudo, ww.pseudo).
  - Obersee (bw.pseudo, chw.pseudo, ew.pseudo, sb.pseudo).

## 3 Results

### 3.1 Stability of the pmv/spmv intercorrelation

The range of deviations attributable to variability in the pmv intercorrelation between simulation repetitions, i.e. Δ{pmv-rmv}, was effectively zero, primarily being due to rounding errors (-5·10^−15^ to 5·10^−15^). Moreover, the range of deviations that resulted from the random sampling of phs across simulation repetitions, i.e. Δ{spmv-rmv}, was small. The largest positive deviation was 0.039, the largest negative deviation was -0.083 for any spmv correlation matrix entry.

### 3.2 Composition of the pseudo reference chronologies

#### 3.2.1 phs approaches

When setting the t-value threshold to 10 (t10) and 15 (t15), respectively, the percentage of generated series classified lay below 1.2% with the phs approach (Table 1). The number of PREF-Constructor runs executed per simulation repetition was very low (often < 3 runs, Table 1). Only the t5 threshold resulted in prefs that were long and well replicated enough to allow for further analysis of their composition.

**Table 1:** Summary statistics for the different simulation settings (approaches) evaluated.

approach	% of generated classified			% of classified correct			No. runs executed			No. of series generated		
	Min	Median	Max	Min	Median	Max	Min	Median	Max	Min	Median	Max
poc_t15_1	19.82	34.67	46.33	71.41	96.76	99.93	13	23	39	6322	6417	6501
poc_t15_0.83	4.19	11.47	19.46	61.47	92.70	100.00	3	18	37	6319	6418	6518
poc_t15_0.67	0.82	3.74	8.53	43.53	94.83	100.00	2	9	31	6314	6418	6503
poc_t20_1	0.85	2.85	6.05	75.00	100.00	100.00	2	7	21	6322	6417	6501
poc_t10_1	67.12	78.38	86.23	57.86	75.63	87.15	15	24	47	6322	6417	6501
poc_t10_0.83	56.22	65.56	76.27	53.03	65.52	77.26	13	23	49	6319	6418	6518
poc_t10_0.67	45.88	53.89	60.56	37.35	50.14	61.56	11	17	40	6314	6418	6503
poc_t5_1	83.34	90.54	92.81	30.31	37.38	47.75	7	10	25	6322	6417	6501
phs_t15	0.00	0.00	0.02	0.00	0.00	100.00	0	0	1	6312	6414	6503
phs_t10	0.09	0.44	1.18	77.59	100.00	100.00	1	2	11	6312	6414	6503
phs_t5	43.42	55.54	60.97	33.36	43.28	54.28	11	16	41	6312	6414	6503

**Fig 2:**
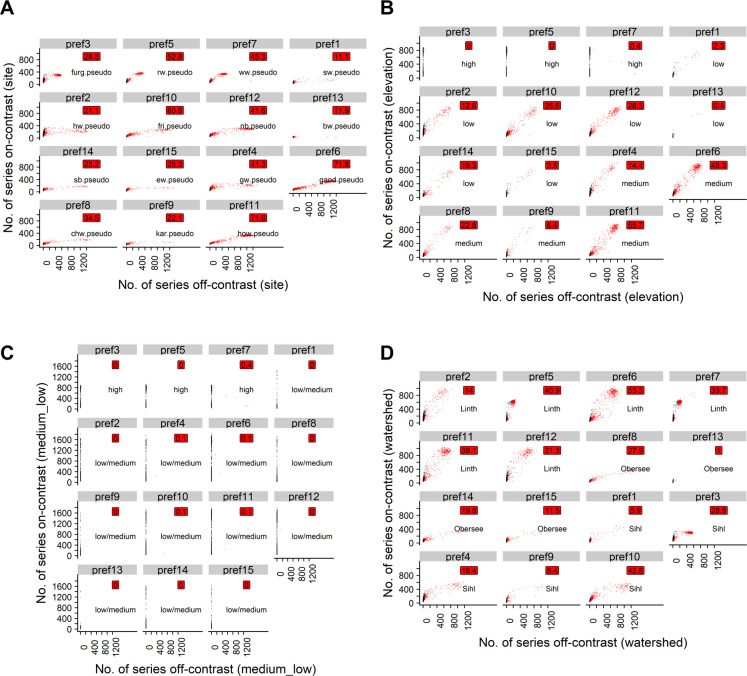
Scatterplots for the phs approach with t5. Black points: Uncontaminated simulation repetitions (<20% off-contrast series). Red points: Contaminated simulation repetitions (>20% off-contrast series). Red labels: Percentage of simulation repetitions in which the respective pref was contaminated. Text labels: The contrast group that the respective pref was attributed to. A) Site contrast; prefs sorted according to elevation band of the real site signals underlying the pseudo site signals used to initialize the respective prefs. B) Elevation contrast; prefs sorted according to elevation. C) Elevation contrast rough (medium and low elevations aggregated); prefs sorted according to elevation. D) Watershed contrast; prefs sorted according to watershed.

However, the t5 prefs were quickly contaminated. By the end of the 2nd PREF-Constructor run, contamination was <20% for all prefs in 95% of the simulation repetitions (Table 2). By the end of the final PREF-Constructor run, pref1 and pref13 were the only prefs that were contaminated by less than 20% in more than 80% of the repetitions (Fig 2A). These were also the shortest and most sparsely replicated prefs overall (Table 2). Contamination was higher for the longer and better replicated prefs. For example, prefs no. 4, 5, 6, 7, 10 and 11 were contaminated by more than 20% off-site series in >40% of the simulation repetitions (Fig 2A).

**Table 2:** 95% percentile of contamination, median length, and median mean replication for the phs approach at t5.

	pref1			pref2			pref3			pref4			pref5		
	Cont.	Leng.	Repl.	Cont.	Leng.	Repl.	Cont.	Leng.	Repl.	Cont.	Leng.	Repl.	Cont.	Leng.	Repl.
run1	11.4	218.0	11.1	5.6	231.0	15.1	4.3	231.0	14.9	11.8	231.0	14.3	7.1	230.0	15.6
run2	15.0	245.0	12.6	11.1	288.0	17.7	12.0	286.0	17.5	20.5	283.0	16.6	17.7	298.0	18.6
run3	20.3	257.0	13.6	24.7	342.0	19.7	28.1	330.0	19.7	37.2	317.0	18.7	36.0	352.5	21.3
run4	27.4	264.0	14.3	42.4	368.5	21.1	43.5	357.0	20.8	51.9	338.0	20.0	45.7	395.0	23.1
run5	30.4	265.5	14.6	54.9	388.0	22.0	52.2	370.0	21.4	61.9	347.0	21.1	50.8	421.5	24.3
run6	31.6	267.0	14.8	64.9	402.5	22.5	57.0	374.0	21.7	69.0	353.5	21.4	53.8	432.0	25.6
run7	32.4	267.5	15.0	70.7	411.0	22.7	59.8	377.0	21.9	74.5	355.5	21.6	55.2	432.5	26.6
run8	32.4	268.0	15.0	75.0	417.0	22.8	62.1	378.0	22.0	77.5	358.0	21.8	56.3	437.5	26.8
run9	32.4	268.0	15.0	78.2	422.5	22.9	63.0	378.5	22.0	79.4	358.5	21.9	57.3	437.5	27.0
run10	33.0	268.0	15.1	80.2	427.0	23.0	63.9	379.0	22.0	81.7	359.0	21.9	57.8	437.5	27.1
run11	33.7	268.0	15.1	81.0	430.0	23.1	64.4	379.0	22.0	82.2	359.0	21.9	58.1	437.5	27.1
	pref6			pref7			pref8			pref9			pref10		
	Cont.	Leng.	Repl.	Cont.	Leng.	Repl.	Cont.	Leng.	Repl.	Cont.	Leng.	Repl.	Cont.	Leng.	Repl.
run1	16.7	233.0	15.9	7.0	230	15.1	15.6	223.5	12.0	15.0	217	9.9	16.3	229	14.3
run2	35.0	303.0	20.1	18.4	286	18.0	27.1	260.0	13.9	20.8	241	11.3	27.5	282	17.1
run3	59.1	356.5	23.6	36.9	335	20.3	45.0	272.0	15.1	32.8	249	12.1	46.7	313	19.3
run4	68.0	384.5	26.2	46.4	358	21.4	56.2	277.5	15.8	43.3	253	12.5	58.3	327	20.6
run5	72.8	393.5	27.8	52.3	367	22.1	65.8	278.5	16.1	49.2	254	12.8	67.3	329	21.4
run6	75.3	394.0	28.4	54.9	372	22.5	71.2	279.5	16.2	51.7	254	12.9	73.5	332	21.7
run7	76.7	395.0	28.6	56.9	372	22.8	73.4	280.0	16.3	53.1	254	12.9	76.8	335	21.8
run8	77.6	397.5	28.9	58.1	372	22.9	75.4	280.0	16.3	53.7	254	13.0	78.0	335	22.1
run9	78.4	397.5	29.0	59.1	372	23.1	75.0	280.0	16.3	55.2	254	13.1	80.3	335	22.2
run10	78.9	397.5	28.9	59.8	372	23.0	75.1	280.0	16.3	55.2	254	13.1	81.0	335	22.2
run11	79.1	398.5	28.9	60.2	372	23.1	75.5	280.0	16.3	56.1	254	13.1	81.6	335	22.2
	pref11			pref12			pref13			pref14			pref15		
	Cont.	Leng.	Repl.	Cont.	Leng.	Repl.	Cont.	Leng.	Repl.	Cont.	Leng.	Repl.	Cont.	Leng.	Repl.
run1	17.0	232.0	15.4	11.1	231.5	15.1	18.2	205.5	6.9	11.1	220.0	12.5	15.2	220.0	11.0
run2	37.2	295.0	19.2	23.7	281.0	18.3	20.6	214.0	7.8	18.4	255.5	14.4	20.0	247.0	12.6
run3	58.7	334.0	22.0	45.3	313.5	20.4	25.0	219.0	8.1	32.6	273.0	15.8	29.5	263.0	13.7
run4	68.8	348.0	23.7	59.9	327.0	21.2	27.0	222.0	8.4	46.9	281.0	16.6	38.5	269.0	14.3
run5	73.5	354.0	24.3	68.6	328.0	21.7	28.6	223.0	8.5	56.3	284.0	17.1	44.5	273.0	14.7
run6	75.8	356.5	24.9	72.9	330.0	21.9	30.3	223.0	8.5	64.8	286.5	17.3	46.1	274.0	15.0
run7	77.6	357.0	25.0	76.4	330.0	22.0	30.6	223.0	8.6	67.1	287.0	17.4	47.0	275.0	15.0
run8	78.7	357.0	25.1	78.2	330.0	22.1	30.8	223.0	8.6	71.2	287.0	17.4	48.1	275.5	15.1
run9	79.4	357.0	25.2	79.6	330.0	22.1	30.8	223.0	8.6	72.0	287.5	17.5	49.2	276.0	15.2
run10	79.8	357.0	25.2	80.4	330.0	22.1	31.1	223.0	8.6	72.7	288.0	17.5	49.5	276.0	15.2
run11	80.0	357.0	25.2	80.8	330.0	22.1	31.5	223.0	8.6	73.3	288.0	17.5	49.5	276.0	15.2

Statistics were calculated for the fully replicated PREF-constructor runs only, i.e. runs that were executed in all 1000 simulation repetitions of the validation process. Full tables including the statistics for lower replicated runs are provided in S3 File.

Regarding contrasts other than the site contrast, prefs initialized with high-elevation signals only attracted phs sampled from high-elevation pseudo signals. Low- and medium-elevation signals, however, were often mixed (Fig 2B). The same was true for signals belonging to different watersheds (Fig 2D, Fig 2 in S2 File, Table 1 in S3 File). Remarkably, in 891 out of 1000 simulation repetitions, 2 full-length prefs were developed, one of which consisted of high-elevation phs and the other of a mixture of medium and low-elevation phs. The rest of the prefs were predominantly short (1–333 years) or at least shorter than 667 years (Table 3).

**Table 3:** Mean number of prefs in the respective length (left) and attractor categories (right).

approach	length					phs/pocs attracted			
	0	1–333	334–667	668–999	1000	0–100	101–500	501–1000	>1000
poc_t15_1	0	6	3	2	4	8	7	0	0
poc_t15_0.83	0	10	3	1	0	13	2	0	0
poc_t15_0.67	1	13	1	0	0	15	0	0	0
poc_t20_1	3	11	1	0	0	15	0	0	0
poc_t10_1	0	2	5	3	6	1	11	2	0
poc_t10_0.83	0	3	6	2	3	3	9	2	1
poc_t10_0.67	0	7	5	1	2	8	5	1	1
poc_t5_1	0	6	6	1	2	6	6	1	1
phs_t15	15	0	0	0	0	15	0	0	0
phs_t10	7	8	0	0	0	15	0	0	0
phs_t5	0	8	4	0	2	9	4	1	1

Attractor category denotes the mean no. of series attracted per pref. For example, in mean (over all 1000 repetitions), there were 8 prefs per repetition that attracted 0–100 pocs with the poc approach at t15 and osr-1.

#### 3.2.2 poc approaches

**Fig 3:**
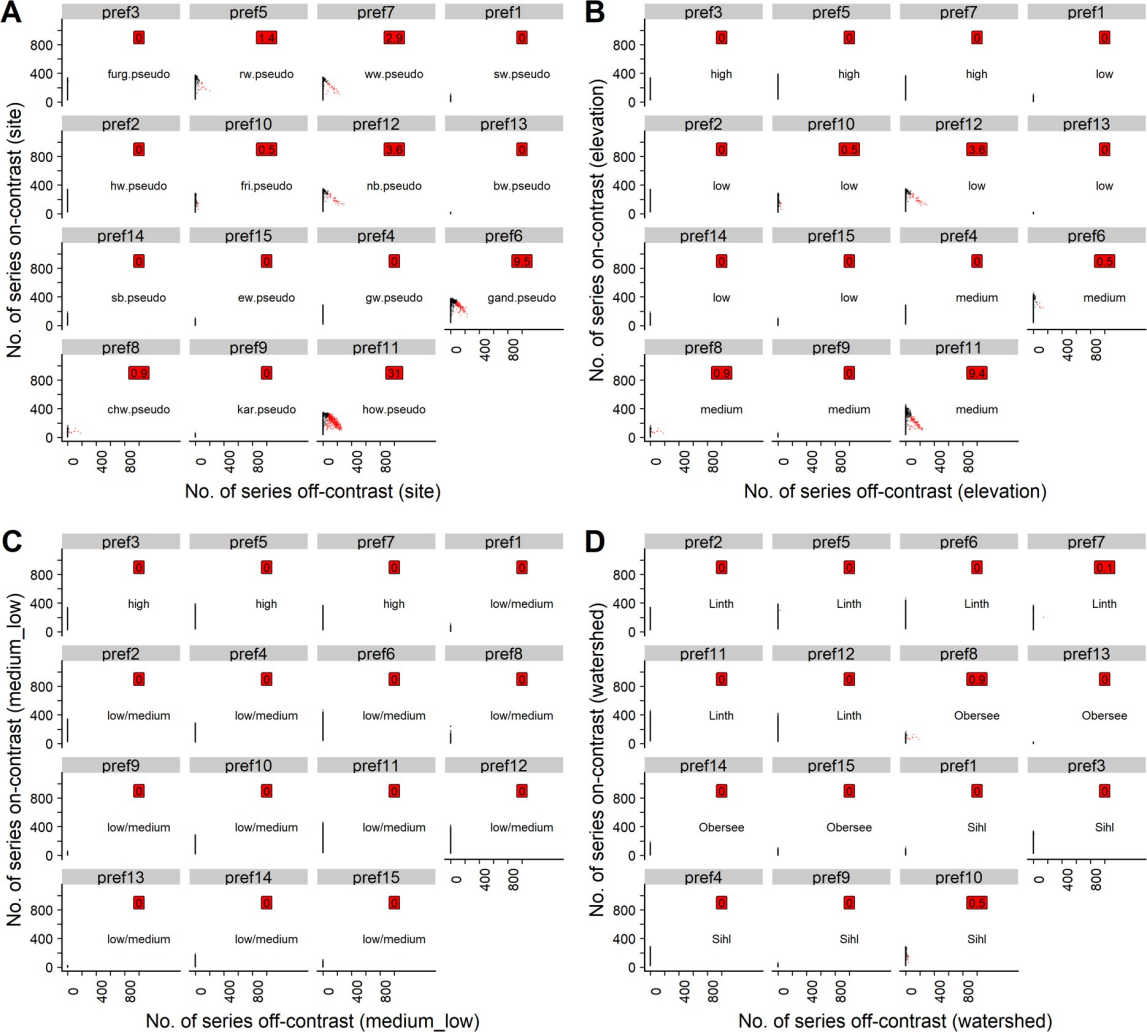
Scatterplots for the poc approach with t15 and osr-1. Black points: Uncontaminated simulation repetitions (<20% off-contrast series). Red points: Contaminated simulation repetitions (>20% off-contrast series). Red labels: Percentage of simulation repetitions in which respective pref was contaminated. For text labels and explanation of the subplots, cf. caption of Fig 2.

*t15 threshold*. When setting the on-site ratio to 1 (osr-1) and the t-value threshold to 15 (t15), between 19.82% and 46.33% of the generated series were classified by the PREF-Constructor algorithm. Of these, between 71.41% and 99.93% were classified correctly (Table 1), depending on the simulation repetition. For those PREF-Constructor runs that were replicated by all repetitions, the contamination was <20% until run no. 6 for all prefs generated (Table 4). Moreover, even when calculating the contamination after the last PREF-Constructor run had been executed, pref6 and pref11 were the only prefs that exhibited a contamination >20% with a notable frequency, i.e. in more than 5% of the repetitions (Fig 3A). For the other prefs, highly contaminated cases (>50%) occurred only rarely (<5% of the repetitions, Table 2 in S3 File).

**Table 4:** 95% percentile of contamination, median length, and median mean replication for the poc approach (t15, osr-1).

	pref1			pref2			pref3			pref4			pref5		
	Cont.	Leng.	Repl.	Cont.	Leng.	Repl.	Cont.	Leng.	Repl.	Cont.	Leng.	Repl.	Cont.	Leng.	Repl.
run1	0	205.0	6.6	0	229.0	13.1	0	227.0	13.0	0.0	226.0	11.1	0.0	228.0	14.5
run2	0	221.0	7.5	0	279.5	15.0	0	277.0	15.2	0.0	267.0	12.9	0.0	289.0	17.0
run3	0	228.0	7.9	0	323.5	16.7	0	327.0	16.5	0.0	302.5	14.1	0.0	351.0	18.5
run4	0	230.0	8.2	0	371.0	17.8	0	372.0	17.6	0.0	332.0	15.1	0.0	407.0	19.7
run5	0	232.0	8.4	0	414.0	18.5	0	414.0	18.6	0.0	360.0	15.9	0.6	461.0	20.8
run6	0	232.5	8.5	0	456.5	19.3	0	452.0	19.2	0.0	387.0	16.4	1.0	514.0	21.4
run7	0	232.5	8.6	0	494.0	19.7	0	496.0	19.6	0.0	407.5	16.6	1.4	569.0	21.9
run8	0	232.5	8.6	0	538.0	20.2	0	534.0	20.0	0.0	427.0	16.9	1.6	625.0	22.4
run9	0	232.5	8.7	0	580.5	20.5	0	567.0	20.3	0.0	448.0	17.2	1.8	676.0	22.9
run10	0	232.5	8.7	0	617.0	20.8	0	603.0	20.7	0.5	454.5	17.5	2.3	727.5	23.2
run11	0	232.5	8.7	0	653.5	21.0	0	641.0	20.8	0.5	461.0	17.7	2.4	779.5	23.5
run12	0	232.5	8.7	0	693.0	21.2	0	674.5	21.1	0.5	470.5	17.8	2.5	823.0	23.8
run13	0	232.5	8.7	0	730.0	21.5	0	705.0	21.5	0.5	474.5	17.9	2.8	871.0	24.2
	pref6			pref7			pref8			pref9			pref10		
	Cont.	Leng.	Repl.	Cont.	Leng.	Repl.	Cont.	Leng.	Repl.	Cont.	Leng.	Repl.	Cont.	Leng.	Repl.
run1	0.0	229.0	14.6	0.0	228.5	13.5	0	211.0	7.8	0	198.0	5.0	0.0	221.0	11.7
run2	0.0	289.0	16.9	0.0	278.0	15.5	0	234.0	8.8	0	210.0	5.7	0.0	263.0	13.4
run3	1.4	348.0	18.7	0.0	331.0	17.1	0	247.0	9.6	0	214.0	6.0	0.0	299.5	14.5
run4	3.0	396.0	20.0	0.0	384.5	18.2	0	255.0	10.2	0	215.0	6.2	0.0	331.0	15.4
run5	5.0	452.5	21.0	0.6	435.0	18.9	0	261.0	10.5	0	215.5	6.3	0.9	357.0	16.2
run6	7.5	508.0	21.6	0.7	476.0	19.8	0	263.5	10.8	0	215.5	6.3	1.0	386.0	16.7
run7	11.0	557.5	22.2	0.8	519.5	20.4	0	264.0	10.9	0	215.5	6.4	1.3	402.0	17.1
run8	14.0	611.0	22.8	1.3	562.5	20.8	0	264.5	11.1	0	215.5	6.4	1.6	424.0	17.4
run9	14.9	659.5	23.2	1.6	598.5	21.3	0	264.5	11.2	0	215.5	6.4	1.7	440.0	17.7
run10	18.5	710.0	23.8	1.8	637.5	21.6	0	264.5	11.2	0	215.5	6.4	1.8	456.5	18.0
run11	22.9	751.0	24.1	2.3	667.0	22.0	0	264.5	11.3	0	215.5	6.4	2.0	468.5	18.2
run12	26.5	792.0	24.4	2.6	698.5	22.1	0	264.5	11.3	0	215.5	6.4	2.2	478.5	18.3
run13	27.9	838.5	24.6	2.8	726.5	22.3	0	264.5	11.3	0	215.5	6.4	2.3	483.5	18.5
	pref11			pref12			pref13			pref14			pref15		
	Cont.	Leng.	Repl.	Cont.	Leng.	Repl.	Cont.	Leng.	Repl.	Cont.	Leng.	Repl.	Cont.	Leng.	Repl.
run1	0.0	229.0	13.8	0.0	230.5	13.5	0	169.0	2.2	0	210.0	8.3	0	203.0	6.5
run2	0.0	283.0	15.9	0.0	280.0	15.6	0	171.0	2.4	0	234.0	9.4	0	219.0	7.4
run3	1.9	332.0	17.6	0.0	326.5	17.3	0	171.5	2.5	0	254.0	10.2	0	224.0	7.9
run4	5.0	376.5	19.0	1.0	374.5	18.2	0	171.5	2.5	0	269.5	10.8	0	228.0	8.2
run5	10.1	420.0	19.7	1.6	418.0	19.2	0	171.5	2.5	0	275.5	11.2	0	229.5	8.4
run6	17.9	460.5	20.6	2.5	457.5	19.7	0	171.5	2.5	0	278.0	11.5	0	230.0	8.5
run7	24.6	496.0	21.1	2.8	487.5	20.4	0	171.5	2.5	0	278.0	11.6	0	230.0	8.6
run8	31.6	524.0	21.5	3.2	530.0	20.6	0	171.5	2.5	0	278.0	11.9	0	230.0	8.6
run9	38.0	553.5	21.8	3.4	569.0	21.0	0	171.5	2.5	0	278.0	12.0	0	230.0	8.6
run10	42.1	579.0	22.2	4.3	603.0	21.4	0	171.5	2.5	0	278.0	12.0	0	230.0	8.7
run11	45.2	597.5	22.4	5.6	634.0	21.6	0	171.5	2.5	0	278.5	12.1	0	230.0	8.7
run12	47.2	611.5	22.6	6.4	663.5	21.9	0	171.5	2.5	0	278.5	12.1	0	230.0	8.7
run13	49.5	625.0	22.8	7.6	690.5	22.1	0	171.5	2.5	0	278.5	12.1	0	230.0	8.7

Statistics were calculated for fully replicated PREF-constructor runs only, i.e. runs that were executed in all 1000 simulation repetitions of the validation process. Full tables including statistics for lower replicated runs are provided in S3 File.

On average, per repetition 4 prefs reached the full length of 1000 years. The majority of the prefs were >333 years and often >500 years long. However, in each repetition, on average 6 of the prefs reached <334 years (Table 3, Table 2 in S3 File). The ranking of shortest to longest pref tended to differ considerably by repetition (e.g. pref1 in certain repetitions was among the longest and in other repetitions among the shortest prefs). However, over all simulation repetitions, certain prefs developed systematically better than others (Table 4, Fig 3 in S2 File, Table 2 in S3 File). Among the frequently longer prefs were no. 2, 3, 5, 6 and 7, with a median length >700 years (calculated at the last PREF-constructor run replicated by all 1000 simulation repetitions, Table 4). The shorter prefs were no. 1, 4, 8, 9, 10, 13, 14 and 15, as reflected by a generally lower median length (<500 years; calculated at the last fully replicated PREF-constructor run, Table 4). These prefs also had a lower replication, i.e. a median mean replication <20 series by the end of the last (13th) fully replicated PREF-Constructor run (Table 4; for full tables of minimum, median and maximum lengths and mean replication, respectively, cf. Table 2 in S3 File).

**Fig 4:**
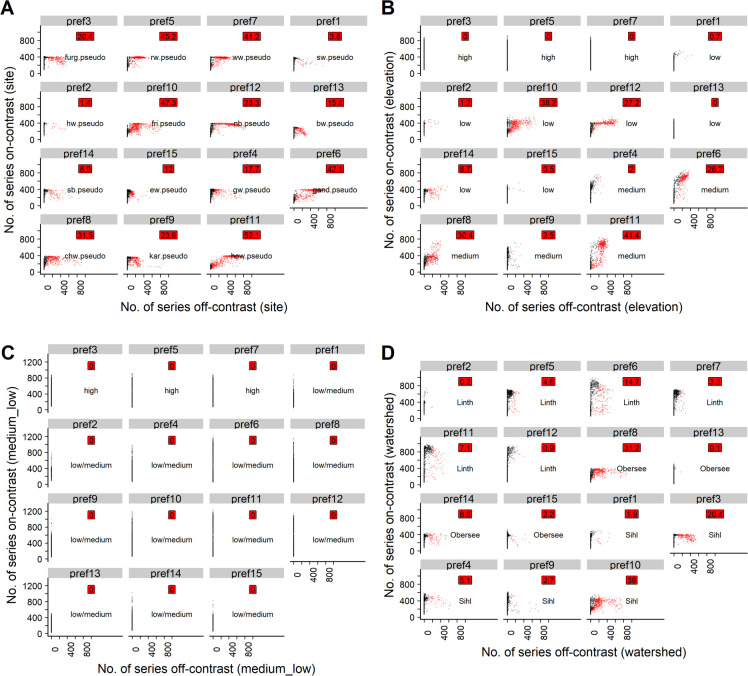
Scatterplots for the poc approach with t10 and osr-1. Black points: Uncontaminated simulation repetitions (<20% off-contrast series). Red points: Contaminated simulation repetitions (>20% off-contrast series). Red labels: Percentage of simulation repetitions in which the respective pref was contaminated. For text labels and explanation of the subplots, cf. caption of Fig 2.

**Fig 5:**
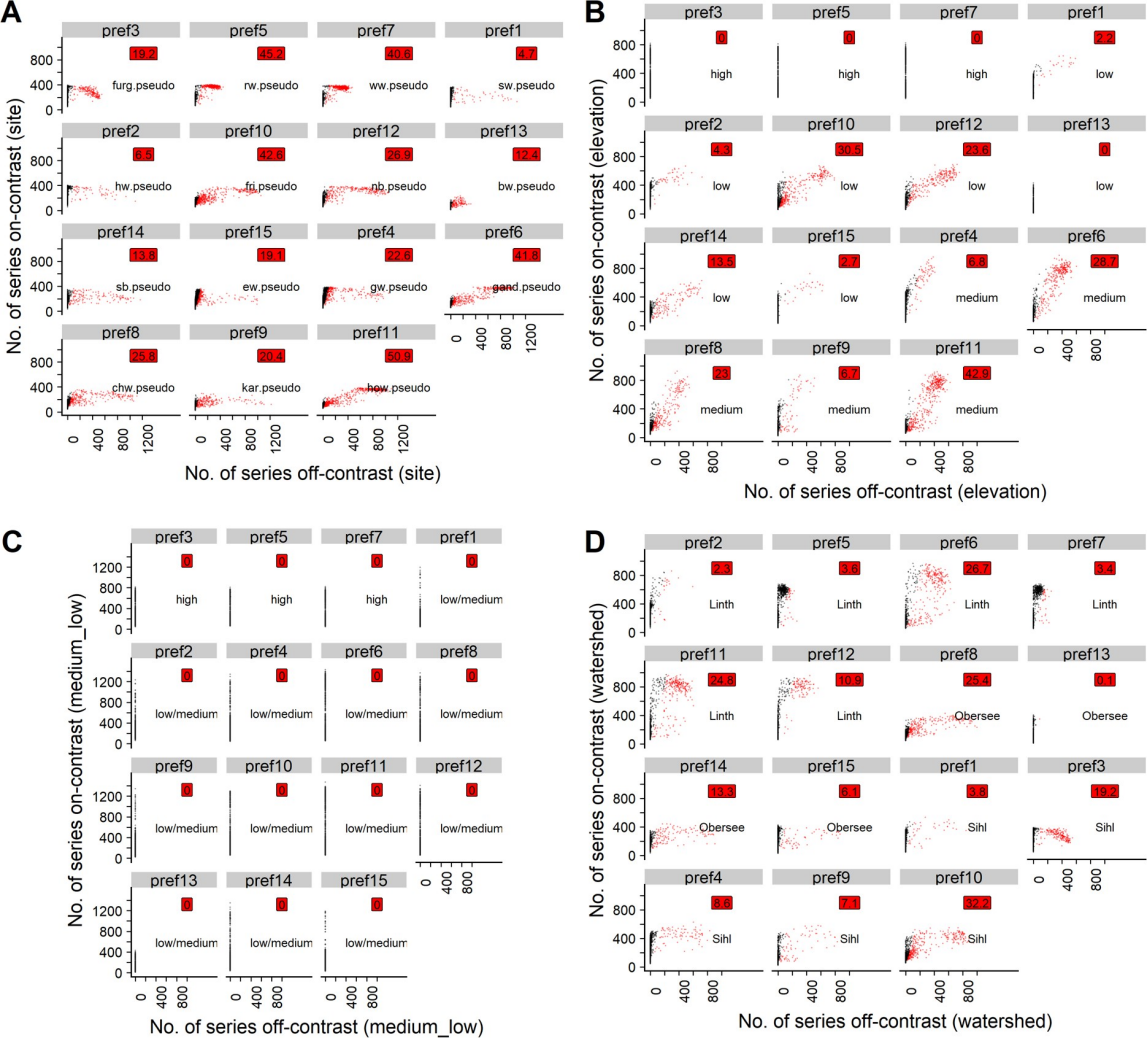
Scatterplots for the poc approach with t10 and osr-0.83. Black points: Uncontaminated simulation repetitions (<20% off-contrast series). Red points: Contaminated simulation repetitions (>20% off-contrast series). Red labels: Percentage of simulation repetitions in which the respective pref was contaminated. For text labels and explanation of the subplots, cf. caption of Fig 2.

**Fig 6:**
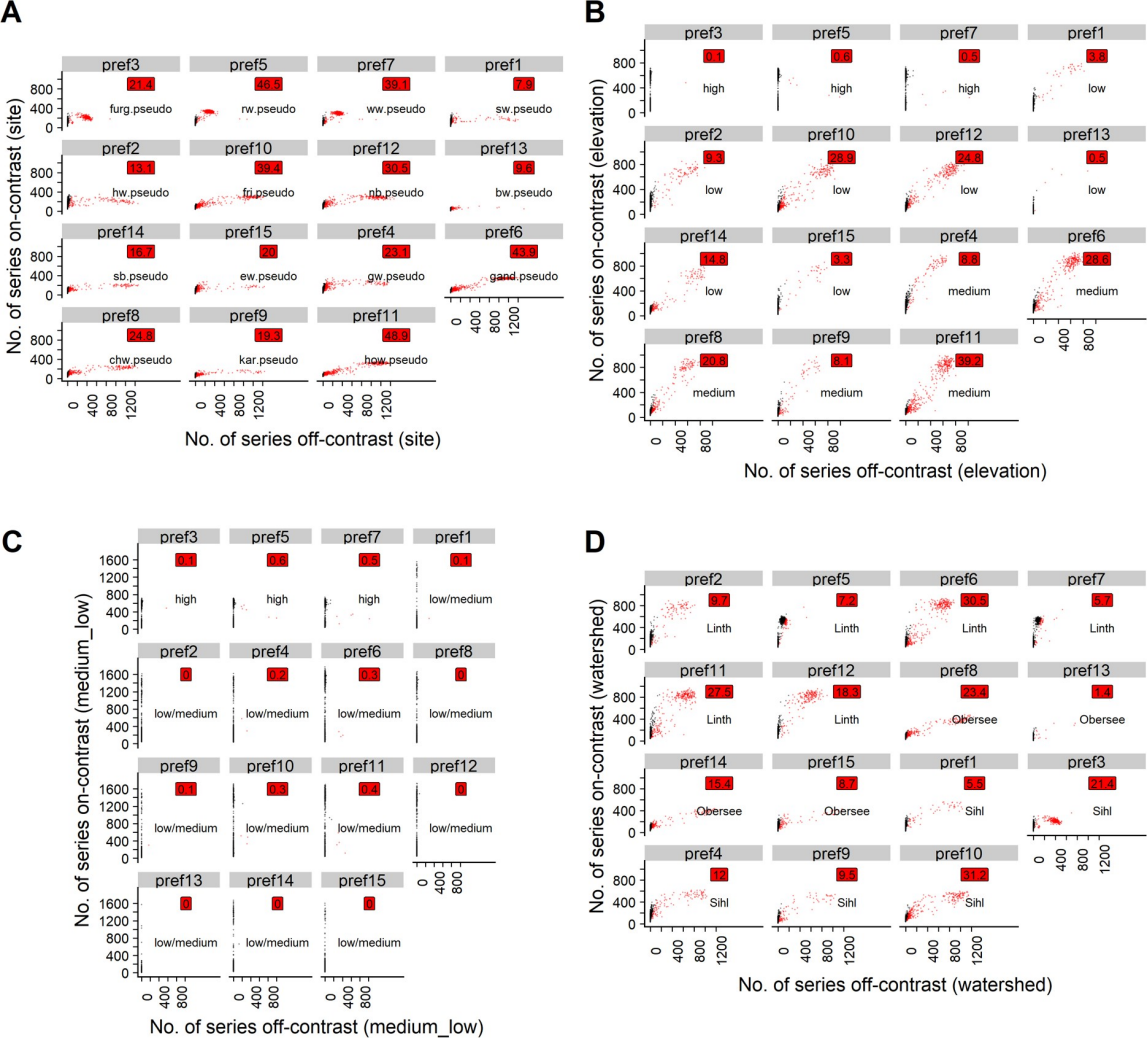
Scatterplots for the poc approach with t10 and osr-0.67. Black points: Uncontaminated simulation repetitions (<20% off-contrast series). Red points: Contaminated simulation repetitions (> 20% off-contrast series). Red labels: Percentage of simulation repetitions in which the respective pref was contaminated. For text labels and explanation of the subplots, cf. caption of Fig 2.

**Fig 7:**
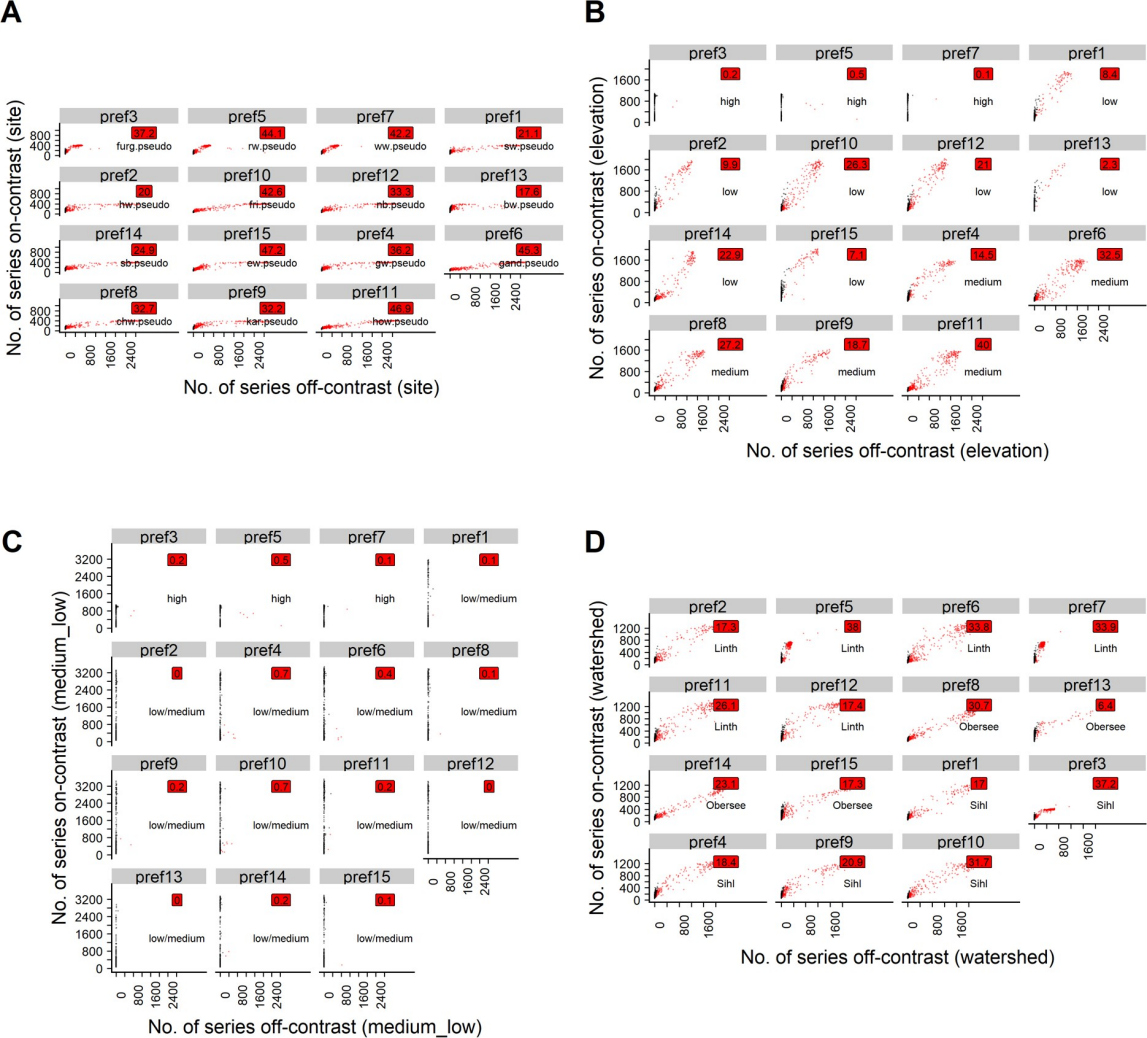
Scatterplots for the poc approach with t5 and osr-1. Black points: Uncontaminated simulation repetitions (<20% off-contrast series). Red points: Contaminated simulation repetitions (>20% off-contrast series). Red labels: Percentage of simulation repetitions in which respective pref was contaminated. For text labels and explanation of the subplots, cf. caption of Fig 2.

Lowering the on-site ratio to 0.83 (osr-0.83) and 0.67 (osr-0.67), respectively, resulted in a drop of classification rates (Table 1). Consequently, fewer PREF-Constructor runs were executed per repetition. Especially with osr-0.67, prefs generally remained short and sparsely replicated (Figs 4–7 in S2 File, Tables 3–4 in S3 File). In extreme cases, e.g. pref13 initialized with bw.pseudo, prefs remained quasi in their initial state (Figs 6–7 in S2 File, Table 4 in S3 File, S4 File).

*t20*, *t10 and t5 threshold*. Short and sparsely replicated prefs resulted also when increasing the t-value threshold to t20 at osr-1, i.e. the maximum on-site ratio (Figs 8–9 in S2 File, Table 5 in S3 File, S4 File). As for t15 at osr-0.67, prefs often remained in their initial state. Thus, no simulations featuring alternative on-site ratios were executed for t20.

For t10 at osr-1, the contamination risk varied considerably between prefs. After the first three PREF-Constructor runs, in 95% of the repetitions most prefs, i.e. no. 1, 2, 3, 4, 5, 7, 8, 9, 10, 13, 14 and 15, exhibited a low contamination (<20%, Table 5). However, in the bulk of repetitions, the majority of prefs were contaminated progressively with every subsequent PREF-Constructor run (Table 5). Nevertheless, usually long (median 453 to 1000 years) and well replicated (median 20 to 28.7) prefs were established with t10 at osr-1 (medians calculated at PREF-constructor run 15, Tables 3 and 5, Fig 4, Table 6 in S3 File, S4 File, Fig 10 in S5 File). Decreasing the on-site ratio for t10 did not affect the length and replication of the prefs as heavily as for t15 (Tables 1 and 3). In summary however, the same observations were made, i.e. the number of correctly classified series decreased the lower the on-site ratio (Table 1, Figs 5, 6, Tables 7, 8 in S3 File, Figs 11–12 in S5 File). Consequently, and especially with osr-0.67, the length and replication of prefs decreased in most repetitions (S4 File).

**Table 5:** 95% percentile of contamination, median length, and median mean replication for the poc approach (t10, osr-1).

	pref1			pref2			pref3			pref4			pref5		
	Cont.	Leng.	Repl.	Cont.	Leng.	Repl.	Cont.	Leng.	Repl.	Cont.	Leng.	Repl.	Cont.	Leng.	Repl.
run1	0.0	233.0	15.3	0.0	235	16.1	0.0	234.0	16.1	0.0	235.0	16.0	0.0	232.0	16.2
run2	0.0	299.0	17.9	0.0	310	19.7	1.2	315.0	19.8	1.6	312.0	19.6	5.0	318.0	19.8
run3	1.5	369.0	19.8	0.5	393	21.8	3.5	400.0	21.7	4.7	390.0	21.7	16.8	403.0	22.1
run4	2.2	424.0	21.3	0.6	467	23.1	8.8	481.0	23.3	9.4	464.0	23.1	28.6	481.5	23.9
run5	3.6	473.0	22.4	0.8	537	24.1	18.6	546.0	24.4	14.7	528.0	24.1	36.3	553.0	24.8
run6	5.4	519.0	23.0	1.0	609	24.7	30.5	614.5	24.9	19.5	586.5	25.1	41.9	611.5	25.8
run7	7.5	565.0	23.6	1.3	680	25.3	40.3	671.0	25.5	27.0	630.5	25.6	44.1	669.5	26.2
run8	8.3	598.5	24.0	1.3	738	25.7	46.9	725.0	25.9	31.3	682.0	26.2	46.3	713.0	26.7
run9	9.1	637.0	24.3	1.3	799	26.1	51.6	769.5	26.2	34.5	718.5	26.5	47.6	745.5	27.2
run10	9.3	665.5	24.6	1.3	859	26.3	54.3	803.5	26.5	36.5	756.5	26.8	48.1	760.0	27.8
run11	9.1	698.0	24.7	1.3	913	26.7	54.8	828.0	26.6	37.2	777.0	27.2	48.8	777.5	28.3
run12	10.8	726.5	24.9	1.3	949	27.0	55.9	838.5	26.8	38.2	816.0	27.4	48.9	782.5	28.5
run13	10.8	756.5	24.9	1.3	1000	27.5	56.4	850.0	26.9	38.9	843.0	27.6	49.0	783.5	28.6
run14	10.8	785.5	25.0	1.3	1000	28.2	56.3	866.5	27.0	39.6	872.0	27.8	49.0	784.0	28.7
run15	10.8	818.0	25.1	1.3	1000	28.3	56.3	876.5	27.0	40.3	888.5	28.0	49.1	784.0	28.7
	pref6			pref7			pref8			pref9			pref10		
	Cont.	Leng.	Repl.	Cont.	Leng.	Repl.	Cont.	Leng.	Repl.	Cont.	Leng.	Repl.	Cont.	Leng.	Repl.
run1	0.0	232.0	16.3	0.0	232.0	16.2	0.0	233.0	15.6	0.0	234.0	14.7	0.0	231.0	16.3
run2	12.2	312.0	20.3	4.9	312.0	19.8	3.7	301.5	18.8	1.4	294.0	17.4	4.2	312.0	19.6
run3	29.8	383.0	22.7	18.3	395.5	22.1	14.3	365.5	20.9	3.8	348.5	19.3	14.2	380.0	22.1
run4	41.3	440.0	24.0	30.8	463.0	23.7	26.7	414.5	22.5	11.3	400.0	20.6	27.4	444.0	23.6
run5	50.7	472.5	25.0	37.8	528.0	24.8	36.6	455.0	23.6	22.0	439.0	21.7	37.5	487.0	24.7
run6	55.7	491.0	25.5	43.8	584.5	25.4	43.6	482.5	24.2	30.6	475.0	22.3	43.5	522.0	25.6
run7	59.6	497.0	25.8	46.6	621.0	25.9	48.4	504.0	24.6	39.2	506.0	22.9	50.1	543.0	26.2
run8	61.7	501.0	26.0	49.0	650.0	26.3	51.6	517.0	24.9	43.9	529.0	23.2	54.3	563.5	26.4
run9	62.6	501.0	26.0	49.4	665.0	26.4	54.9	526.5	25.1	47.4	551.0	23.5	58.2	581.5	26.7
run10	63.2	503.5	26.2	50.2	668.0	26.7	57.6	539.0	25.2	48.6	575.0	23.6	60.2	598.0	26.9
run11	63.4	503.5	26.2	50.2	668.5	26.8	58.0	541.5	25.2	51.0	589.0	23.8	61.3	613.5	27.1
run12	63.5	503.5	26.2	50.3	668.5	26.9	58.5	546.5	25.2	53.4	606.5	23.8	61.7	620.5	27.3
run13	63.6	503.5	26.3	50.3	668.5	27.0	58.9	550.0	25.2	53.7	616.0	23.9	62.0	630.0	27.2
run14	63.7	503.5	26.3	50.3	668.5	27.0	58.9	552.5	25.2	54.5	624.5	24.0	62.1	631.0	27.4
run15	63.6	503.5	26.3	50.4	668.5	27.0	59.1	552.5	25.3	54.6	637.0	24.0	62.1	632.0	27.5
	pref11			pref12			pref13			pref14			pref15		
	Cont.	Leng.	Repl.	Cont.	Leng.	Repl.	Cont.	Leng.	Repl.	Cont.	Leng.	Repl.	Cont.	Leng.	Repl.
run1	0.0	234.0	16.2	0.0	235.0	16.1	0.0	225.0	12.5	0.0	233.0	15.8	0.0	230.0	15.4
run2	16.6	314.0	20.3	8.0	312.0	20.0	0.0	267.5	14.3	0.0	301.0	18.8	1.0	293.0	18.1
run3	38.3	384.0	22.9	21.1	390.0	22.3	1.8	303.0	15.8	1.6	370.5	20.9	2.0	357.0	19.8
run4	47.7	446.0	24.5	34.8	453.0	23.7	4.0	333.0	16.8	2.7	431.0	22.2	4.3	417.0	21.4
run5	53.6	487.5	25.9	46.2	492.5	24.5	7.7	359.5	17.7	4.7	488.0	23.2	6.3	473.5	22.3
run6	57.8	508.5	27.2	52.2	526.0	25.1	15.5	381.0	18.2	8.8	529.0	24.1	9.5	526.5	23.2
run7	59.8	516.0	27.9	56.4	543.0	25.4	20.3	400.5	18.6	13.4	571.5	24.5	12.1	572.0	23.9
run8	61.6	521.0	28.3	60.2	550.5	25.7	25.3	414.5	18.8	19.5	605.5	24.9	13.4	610.0	24.4
run9	62.7	521.5	28.6	61.6	556.0	25.8	27.8	425.5	19.1	23.4	640.0	25.0	16.0	649.0	24.8
run10	63.3	521.5	28.7	61.9	558.0	25.8	30.3	433.0	19.3	29.6	671.0	25.3	17.8	683.0	25.2
run11	63.6	522.5	28.7	61.9	561.0	25.9	34.1	439.5	19.5	33.6	701.0	25.3	20.2	717.5	25.5
run12	63.7	522.5	28.7	61.9	564.0	25.9	36.4	443.0	19.7	35.7	728.5	25.5	22.7	756.5	25.7
run13	63.8	522.5	28.7	61.9	564.0	25.9	38.3	448.0	19.8	37.1	754.5	25.4	24.2	800.0	25.8
run14	64.0	522.5	28.7	61.9	564.0	25.9	40.8	449.0	19.9	37.4	780.0	25.5	24.9	824.0	26.1
run15	64.1	522.5	28.7	61.9	564.0	25.9	42.5	453.0	20.0	38.4	809.0	25.5	26.5	865.5	26.4

Statistics were calculated for fully replicated PREF-constructor runs only, i.e. runs that were executed in all 1000 simulation repetitions of the validation process. Full tables including statistics for lower replicated runs are provided in S3 File.

A very high contamination risk was observed when lowering the threshold to t5, even while keeping the on-site ratio at its maximum, i.e. osr-1 (Tables 1, 3 and 6, Fig 7, Table 9 in S3 File, S4 File, Fig 13 in S5 File). Thus, for the poc approach at t5, the simulations featuring lower on-site ratios were omitted.

**Table 6:** 95% percentile of contamination, median length, and median mean replication for the poc approach (t5, osr-1).

	pref1			pref2			pref3			pref4			pref5		
	Cont.	Leng.	Repl.	Cont.	Leng.	Repl.	Cont.	Leng.	Repl.	Cont.	Leng.	Repl.	Cont.	Leng.	Repl.
run1	0.0	235	16.1	0.0	235.0	16.2	0.0	234.0	16.1	0.0	235	16.0	0.0	232.0	16.2
run2	35.8	317	20.9	32.2	313.0	20.6	35.0	323.5	20.7	45.8	321	21.2	38.8	329.0	20.9
run3	60.0	356	22.2	59.7	369.5	22.0	49.7	397.0	22.2	69.9	356	22.4	52.1	398.5	22.6
run4	72.5	359	22.3	74.0	373.5	22.3	55.8	435.0	22.8	79.0	357	22.8	57.2	419.5	23.6
run5	78.7	359	22.3	79.8	373.5	22.4	58.5	443.5	22.9	83.3	357	22.8	59.5	421.0	23.8
run6	83.2	359	22.3	83.9	373.5	22.4	60.1	443.5	23.0	85.4	357	22.8	60.9	421.0	23.8
run7	84.1	359	22.4	84.7	373.5	22.4	61.1	443.5	23.0	86.2	357	22.8	61.8	421.0	23.8
	pref6			pref7			pref8			pref9			pref10		
	Cont.	Leng.	Repl.	Cont.	Leng.	Repl.	Cont.	Leng.	Repl.	Cont.	Leng.	Repl.	Cont.	Leng.	Repl.
run1	0.0	232	16.3	0.0	232.0	16.2	0.0	233.5	16.0	0.0	236.0	16.0	0.0	231	16.3
run2	63.4	322	21.9	37.6	324.0	20.9	50.0	315.0	21.6	40.7	316.0	21.4	57.3	319	21.6
run3	77.3	343	23.2	51.1	389.0	22.8	74.1	340.0	22.5	62.4	346.5	22.7	75.1	342	22.8
run4	82.9	343	23.4	56.7	401.0	23.4	81.2	340.0	22.5	73.9	349.0	23.0	82.0	343	23.1
run5	85.6	343	23.4	58.9	403.5	23.6	84.4	340.0	22.6	81.1	350.0	23.1	84.6	343	23.1
run6	86.9	343	23.5	60.3	403.5	23.6	86.1	340.0	22.6	84.4	350.0	23.1	86.3	343	23.1
run7	87.3	344	23.5	61.4	403.5	23.6	86.7	340.0	22.6	84.6	350.0	23.1	86.9	343	23.1
	pref11			pref12			pref13			pref14			pref15		
	Cont.	Leng.	Repl.	Cont.	Leng.	Repl.	Cont.	Leng.	Repl.	Cont.	Leng.	Repl.	Cont.	Leng.	Repl.
run1	0.0	234.0	16.2	0.0	235.0	16.1	0.0	235.0	16.0	0.0	233.0	16.1	0.0	231.5	16.3
run2	60.6	323.5	22.1	50.4	318.5	21.3	22.5	311.0	20.6	46.5	312.0	20.9	40.9	314.0	21.4
run3	77.1	343.5	23.6	72.3	358.0	22.5	38.5	366.5	22.5	70.8	339.5	22.1	63.0	354.0	23.1
run4	82.8	344.0	23.9	80.9	358.0	22.6	47.9	381.0	23.1	80.3	340.0	22.3	71.8	363.5	23.8
run5	85.6	344.0	23.9	84.3	358.0	22.7	52.4	388.0	23.3	83.8	340.0	22.3	79.6	369.5	23.9
run6	87.0	344.0	23.9	86.0	358.0	22.7	53.4	390.0	23.3	86.0	340.0	22.3	82.3	370.0	24.0
run7	87.3	344.0	23.9	86.6	358.0	22.7	52.4	391.0	23.4	86.3	340.0	22.3	82.7	370.0	24.1

Statistics were calculated for fully replicated PREF-constructor runs only, i.e. runs that were executed in all 1000 simulation repetitions of the validation process. Full tables including statistics for lower replicated runs are provided in S3 File.

For all t-value thresholds and on-site ratios investigated, grouping the pseudo signals according to the elevation bands of the underlying real sites revealed that prefs initialized with pseudo signals from high elevation sites did generally not attract poc series that were sampled from medium- or low-elevation pseudo signals (Figs 4C, 5C, 6C and 7C). However, prefs frequently attracted pocs from watersheds other than the pseudo site signal used to initialize the respective pref (Figs 4D, 5D, 6D and 7D).

## 4 Discussion

### 4.1 Adequacy of the simulation model for investigating the contamination risk of reference chronologies

This study assesses a fundamental assumption of dendo-provenancing, i.e. that regional reference chronologies reflect regional tree-growth. Although initially plausible, a systematic evaluation of this assumption is needed prior to dendro-provenancing because non-regional series (e.g. from rafted timbers) may contaminate references (c.f. Introduction). Here, simulating the process of establishing historical reference chronologies was chosen to assess the degree to which local/regional signals can be retained. Algorithmically, pseudo references were established using different thresholds for the minimal similarity required for a given series to be included in the respective reference.

This approach’s success depends on how accurately the site-specific, elevation-specific and regional (dis-)similarities of tree growth in the study area are modelled. The covariance matrix of the real site chronologies (i.e. the 15 Norway spruce site chronologies) represents said growth (dis-)similarities. Hence, the simulated pseudo site signals must evince a covariance matrix that closely reflects the covariance matrix of the real chronologies. Specifically, quasi no deviation was found between the covariance matrix entries of the real chronologies and the simulated pseudo signals (i.e. Δ{pmv-rmv}). Moreover, the pseudo chronologies (sampled from the pseudo signals) consistently reproduced the covariance matrix of the real chronologies in all replications of the simulation (c.f. small range of Δ{spmv-rmv}, Results section). Thus, the 1000 randomly sampled pseudo datasets essentially reflect the same set of site signals. Consequently, these datasets can be used to assess the risk of signal contamination in the study area.

### 4.2 Implications and benefits for dendro-provenancing

For the study region, results indicate that the regional signal of reference chronologies is highly susceptible to contamination. This problem may also be acute in other regions but has received little attention in previous research [5–14, 18–21]. Especially, the contamination risk rises if object chronologies (i.e. the basic components of reference chronologies) already represent a mixed site signal. In the simulation, this was investigated by replacing one or two of the six series that entered a pseudo object chronology with an off-site series (i.e. pocs with osr-0.83 and osr-0.67, respectively). Lowering the on-site signal ratio in the pseudo object chronologies diluted their original pseudo site signal and resulted in a quick decrease of the length and replication of the pseudo reference chronologies constructed using such mixed signal object chronologies.

In the context of sampling real timbers from historical objects, the assumption of an unmixed site signal (i.e. osr-1) seems too optimistic [33]. Realistically, imported timbers occasionally will enter object chronologies [39]. Unfortunately, the simulation was not robust when off-site series were incorporated in pseudo object chronologies. Hence, researchers are best advised to scrutinize the assumption of local timber supply for each object investigated when attempting to establish object chronologies for dendro-provenancing. For the area studied here, reference chronologies that reflect local to regional growth seem procurable only if unmixed object chronologies are used.

Alternatively, establishing regional reference chronologies using single ring-width series seems ineffective and bears a high contamination risk. The pseudo reference chronologies established with the single series (phs) approach were quickly contaminated. In the median, three quarters of the pseudo historical series were either unclassified or wrongly classified (Table 1). Moreover, the pseudo site signal was too weak in the pseudo historical series, thus the t-value threshold could not be raised above t ≥5. Hence, at least for small-scale studies like [15], establishment of reference chronologies and provenancing based on single ring-width series is likely to be highly problematic.

A t-value threshold of t ≥15 was necessary for the robust construction of long and uncontaminated pseudo reference chronologies. This is an extreme restriction for the minimal similarity required for valid matches. In the literature, t ≥15 is an uncommon threshold: often, researchers applied more or less rigorously fixed thresholds between t ≥9 and t ≥11 [7, 8, 40–43]. Some studies even considered t-values <9 to narrow down the area of provenance [44–47]. Strictly speaking, the t-value thresholds are not directly comparable between different studies. Firstly, there exist species specific differences in between series similarity [20, 22]. Yet, these are negligible for illustrating the strict restrictions for the minimal between series similarity that are defined by the requirement t ≥15 in this study. Secondly, the t-value is influenced by preprocessing and the overlap over which the correlation coefficient is calculated [48, 49]. However, the most commonly used t-value calculation methods involve transformations that enhance the high-frequency signal [17, 18]. Thus, the t-value for a correlation coefficient still provides a good approximation of the similarity between ring-width series that was deemed sufficient for a valid match between reference and candidate series in the respective studies cited above (i.e., [7, 8, 40–47]).

The simulation approach that was developed in the present study shows that, even with t ≥15 and unmixed pseudo object chronologies, only one third of the pseudo object chronologies in median was correctly classified (i.e. 34.67% classified to a pseudo reference chronology, of those 96.76% to the correct pseudo reference chronology; Table 1). Lower t-value thresholds evinced a higher risk of establishing contaminated pseudo reference chronologies. For example, although the t ≥10 threshold exhibited a low contamination risk for establishing pseudo reference chronologies in the majority of the repetitions of this simulation study, there were several repetitions in which pseudo reference chronologies that were uncontaminated in most other repetitions were severely contaminated. Particularly, variability was extremely high among the 5% most contaminated simulation repetitions. Thus, no t-threshold can be considered “secure”. Even for t ≥15 with unmixed pseudo object chronologies, contaminated pseudo reference chronologies resulted in rare cases.

Using the best match to extend pseudo reference chronologies represents only one implementable classifier, i.e. the One-Nearest-Neighbor classifier based on t-values for the Pearson’s correlation coefficient (c.f. Methods section). The choice of this classifier over other possibilities was supported by the results of [15]. In future applications, the current approach could be extended by implementing other time series classifiers and/or measures of proximity in the PREF-Constructor algorithm [34].

Regardless of the specific classifier, distance or similarity measurement in use, a simulation approach offers the means for assessing the level of statistical proximity necessary for lowering the risk of establishing contaminated reference chronologies within a given dataset. The t-thresholds determined here are neither universally valid nor directly transferable to other study regions. Because computational and hardware resources were limited, thresholds had to be set for t-values and overlap. Preferably, the calculation would have been continuous. In any real dendro-provenancing study, the dataset displays other clusters of between ring-width series similarities [50–53]. In ideal circumstances, the between-sites signal differences in an area are pronounced enough to allow for site-specific similarity clusters. The range of t-value thresholds that capture the respective level of similarity necessary to establish site-specific, elevation-specific or regional reference chronologies, respectively, should be investigated independently for each study region. This is only possible via simulation, but it seems to have been largely disregarded up to now [5–14, 54].

Moreover, thresholds or t-value ranges that were determined based on simulation require re-evaluation when geographically expanding the dataset of a study region or when investigating other tree species. On the one hand, the pseudo signals for Norway spruce generated here appear disparate when applying the t ≥15 threshold. For neighboring sites, however, narrowing the sampling grid by adding new site chronologies may gradually dissolve the between-site signal differences. On the other hand, certain signal differences may turn out to be more robust and may persist even when new data are added. For example, pronounced elevation specific growth signals have been reported by several studies [55–60]. In the simulation presented here, high-elevation and medium- to low-elevation pseudo site signals proved robust and were separated even when using single series (phs approach at t ≥5) or mixed signal object chronologies (poc approach at t ≥10 and osr-0.67).

## 5 Conclusions

Simulation is paramount for future dendro-provenancing studies. In any study region, the contamination risk of local reference chronologies is unknown *a priori*, since the site provenance of historical timbers remains ambiguous. Thus, this risk can be objectively assessed by simulation only.

In the dataset studied here, uncontaminated pseudo reference chronologies were established only with very strict settings for the t-value and on-site ratio thresholds. Consequently, a high minimal similarity between the object chronologies that enter a reference chronology is required. Moreover, these object chronologies must represent unmixed local site signals.

The approach presented here can be extended in several ways: For example, the input covariance matrix of the real sites could be replaced by any covariance matrix. Also, the thresholds for t-values, on-site ratio and overlap may be replaced by continuous inputs. Also, other classifiers than the current One-Nearest Neighbor classifier could be implemented. However, such developments require high-performance programming techniques and/or hardware. These were beyond the resources available to the author and also beyond the scope of this paper.

Another valuable path for future research would focus on validating or revising the simulation model presented here. For all of the 1000 simulated years, the same intercorrelation structure between the pseudo site signals was assumed. In reality, some temporal variability in the intercorrelation between sites is likely to occur, which could be reflected in the model but was ignored here for the sake of simplicity.

In addition to elaborating the simulation model, it could be extended to incorporate multivariate tree-ring time series of proxies such as wood-density or of stable isotopes like δ^18^O and δ^13^C [61–64]. Multivariate approaches were shown to increase cross-dating success [61, 62]. Thus, such an approach is likely to enhance the spatial resolution of dendro-provenancing and lower the risk of local reference chronology contamination.

Without an adequate evaluation of the contamination risk of reference chronologies, the basis of dendro-provenancing remains inscrutable. The approach presented here is a first and not necessarily the most practicable solution. The depicted avenues of future research hopefully spur methodological innovations in the field that will lead to a more elaborated simulation tool for practical application in dendro-provenancing.

## Supporting information

S1 File R package. *Ad hoc* pseudo.series package.
(RAR)

S2 File Additional Figs 1–9. (RAR)

S3 File Additional Tables 1–9. (RAR)

S4 File Additional details on prefs established with poc approaches. (PDF)

S5 File Additional Figs 10–13. (RAR)
