# Supplementary material for: Assessing site signal preservation in reference chronologies for dendro-provenancing
Source: PLoS One. 2020 Sep 24;15(9):e0239425. doi: 10.1371/journal.pone.0239425 (PMC7514070; doi:10.1371/journal.pone.0239425)
Supplement: S4 File — (PDF) [file pone.0239425.s004.pdf]

S4

Additional details on prefs established with poc approaches

- **t15 threshold at osr-0.83 and osr-0.67**

Lowering the on-site ratio to 0.83 (osr-0.83) and 0.67 (osr-0.67), respectively, decreased the number of PREF-Constructor runs executed per simulation repetition (Table 1). Concurrently, the number of classified series dropped, whereas the percentage of correctly classified series remained relatively stable compared to osr-1. Only for osr-0.67, the minimum of correctly classified series decreased notably. For t15 at osr-0.83, by the end of the 13th PREF-constructor run, most prefs exhibited a median pref-length <400 years (S2 Table 3). However, only in 834 out of 1000 simulation repetitions the PREF-constructor algorithm was executed until run 13. In most repetitions, prefs (especially prefs no. 1, 8, 9, 13, 14, and 15) remained short (median pref-length <250 years) and exhibited a median mean replication <15 (S2 Table 3, S3 Figs 4, 5). When lowering the osr to 0.67, prefs no. 6, 10, 11 and 12 were the only prefs that developed longer (length >250 years) and better replicated (median mean replication >10 series per year) prefs more frequently. The rest of the prefs remained short and sparsely replicated in most repetitions (S2 Table 4, S3 Figs 6, 7).

- **t20 threshold**

Increasing the t-value threshold from t15 to t20 at osr-1 resulted in a drop in PREF-Constructor runs executed. The percentage of generated series being classified was very low (at maximum 6.05%, Table 1). Still, the vast majority of classifications was correct. Thus, the resulting prefs were generally uncontaminated but frequently short (length <250 years) and sparsely replicated (median mean replication <10 series per year; S2 Table 5, S3 Figs 8, 9).

- **t10 threshold**

Between 67.12% and 86.23% of the generated series were classified with the t-value threshold set to 10 (t10) and the on-site ratio set to 1 (osr-1). Of these classified series, at minimum 57.86% and at maximum 87.15% were classified correctly per simulation

repetition (Table 1). The contamination level varied considerably by pref (Table 5). In 95% of the repetitions, prefs no. 1, 2, 3, 4, 5, 7, 8, 9, 10, 13, 14 and 15 were contaminated by less than 20% off-site series by the end of the 3rd PREF-Constructor run, respectively. For prefs no. 1, 2, 14 and 15, the contamination even was <20% until at least the 9th classification run in 95% of the repetitions. Still, only for pref1 and pref2 the contamination remained <20% until the final run in >95% of the repetitions (Fig 4A).

Differences in pref-length and replication were substantially less pronounced than with the t15 threshold (Table 5). On average, 6 prefs reached full length whereas in total 9 out of 15 prefs were >667 years. Also, the number of series attracted per pref varied less than for the t15 threshold. That is, 11 out of 15 prefs attracted >100 and <500 pocs (Table 1). The median length lay between 453 and 1000 years, the median mean replication between 20 and 28.7 series per year (calculated by the end of the last PREF-constructor run executed in all simulation repetitions, i.e. run 15).

Decreasing the on-site ratio did not affect the number of runs executed as severely as for the t15 threshold (Table 1). Basically, the same observations were made, however, i.e. the number of classified series decreased the lower the on-site ratio (Figs 5, 6). In contrast to the t15 threshold, the percentage of generated series classified remained >45%. At the same time, however, the percentage of correctly classified series decreased notably when lowering the on-site ratio. For example, the minimum of correctly classified series decreased from 57.86% (osr-1) to 53.03% (osr-0.83) to 37.35% (osr-0.67). At an on-site ratio of 0.83 the replication and lengths of most prefs were slightly lower than compared to osr-1. The median pref-length lay between 290 and 761 years; the median mean replication between 14.9 and 27.6 (calculated by the end of the last fully replicated PREF-constructor run; S2 Table 7, S3 Fig 11). Reducing the on-site ratio even further (osr-0.67) resulted in a sudden decline of pref-length and median mean replication per year, i.e. pref-lengths between 244 and 486 years; median mean replication between 11.1 and 25.2 (calculated by the end of the last fully replicated PREF-constructor run; S2 Table 8, S3 Fig 12).

- **t5 threshold**

Between 83.34% and 92.81% of the series generated were classified with t5 and osr-1. However, at maximum, 47.75% were classified correctly (Table 1). Consequently, all prefs were contaminated (>20% off-site series) by the second run of the PREF-Constructor already (Table 6). Pref-lengths and median mean replication were equally distributed among prefs (Table 6, S2 Table 9). However, only 2 prefs developed full pref-length. The rest was predominantly of short (1-333 years) or medium length (334-667 years, Table 3).

As stated, on average, 2 full-length prefs were developed per simulation repetition. Similar to the phs approach with t5, in 770 out of 1000 repetitions one out of two full-length prefs consisted of high-elevation pocs whereas the other full-length pref consisted of medium-low elevation pocs. In contrast, the signal mixture between different watersheds was considerable (Fig 7D).

No simulations were calculated for on-site ratios lower than 1 for the t5 threshold, as the contamination by off-site series was frequently >20% for repetitions conducted with osr-1 (Fig 7A, S2 Table 9, S3 Figs 13).
